# Online commercial sex-seeking among female sex workers in south China: a cross-sectional study

**DOI:** 10.1186/s12879-023-08722-x

**Published:** 2023-10-19

**Authors:** Peizhen Zhao, Wenqian Xu, Rouxuan Ye, Yijia Shi, Cheng Wang

**Affiliations:** 1 Dermatology Hospital, Southern Medical University, Guangzhou, 510095 China; 2 Southern Medical University Institute for Global Health, Guangzhou, China; 3 School of Public Health, Southern Medical University, Guangzhou, China; 4 Department of Biostatistics, Bioinformatics & Biomathematics, Georgetown University, Washington, DC USA; 5 Department of Health, Behavior and Society, Johns Hopkins Bloomberg School of Public Health, Baltimore, MD USA

**Keywords:** Online commercial sex‑seeking, Female sex workers, Sexually transmitted diseases, Sexual and reproductive health

## Abstract

**Background:**

Online communication platforms have the potential to facilitate commercial sex among female sex workers (FSW), increasing the risk of contracting sexually transmitted diseases (STD). This study aimed to describe the patterns of online commercial sex-seeking and examine the associated factors among FSW in China.

**Methods:**

A venue-based cross-sectional study was conducted in five cities in Guangdong Province, China, between April and October 2020. Data on socio-demographic characteristics, sexual behaviors, and online commercial sex-seeking patterns were collected through face-to-face interviews. Venous blood and urine samples were collected for STD testing. Univariate and multivariable logistic regressions were used to explore the factors associated with online commercial sex-seeking.

**Results:**

A total of 1155 FSW were recruited in physical venues for this study. Among them, 33.42% reported ever using online applications to seek commercial sex. The prevalence of HIV, syphilis, gonorrhea, chlamydia, and STD was 0.26%, 1.30%, 4.40%, 15.54%, and 18.39%, respectively, among FSW who had ever used both physical and online venues to seek commercial sex, which was higher than among FSW who had never sought commercial sex online. Multivariable logistic regression indicated that FSW who used online platforms to seek commercial sex were more likely to have STD (adjusted odds ratio (a*OR*) = 1.48, 95%CI: 1.05–2.09), experience unintended pregnancies due to commercial sex (a*OR* = 1.78, 95%CI: 1.21–2.62), be diagnosed as infertile (a*OR* = 3.20, 95%CI: 1.42–7.21), and undergo abortions (a*OR* = 1.69, 95%CI: 1.29–2.20).

**Conclusion:**

A significant proportion of FSW who practiced in physical venues in China engaged in seeking commercial sex online, and this behavior is positively correlated with both STD and reproductive health outcomes. Given the high prevalence of online sex-seeking, it is crucial to provide a wide range of internet-based healthcare interventions and reproductive health services to Chinese FSW.

## Background

Female sex workers (FSW) bear a disproportionate burden of sexually transmitted diseases (STD), particularly in low- and middle-income countries, including China [1]. The global prevalence of infection among FSW with human immunodeficiency virus (HIV), chlamydia, gonorrhea, and syphilis ranges from 11.6 to 12.0%, 4.0–15.0%, 1.0–11.0%, and 1.5–42.1%, respectively [2–4]. In China, the corresponding prevalence rates among FSW are 0.2–0.8%, 5.9–25.7%, 16.4–17.3%, and 2.1–13.5% [5–10], are higher than the general population (HIV: 4.2/100,000; chlamydia: 55.3/100,000; gonorrhea: 9.1/100,000, and syphilis: 34.0/100,000) [11, 12].

Online commercial sexual activities are becoming increasingly common among FSW. Previous studies conducted in Europe and Canada have indicated that approximately 10% and 33.9% of FSW, respectively, utilize the internet to seek commercial sex [13, 14]. The prevalence of online commercial sex-seeking among FSW can be attributed to its convenience, discretion, accessibility, safety, and the ease of expanding sexual networks. However, this involvement can lead to an increased likelihood of engaging in condomless sex and having multiple partners compared to FSW who had not sought commercial sex online that facilitate STD transmission [15–19]. The risks associated with these behaviors are further compounded by the ongoing criminalization and stigmatization of FSW, which create barriers hindering their access to health services [20]. Nevertheless, the prevalence of online commercial sex-seeking and its associated factors among Chinese FSW remains unclear. Hence, the objective of this study is to elucidate the prevalence of HIV/STI and reproductive health outcomes, the patterns of online commercial sex-seeking and examine the factors related to this behavior among FSW in China.

## Methods

### Study sites

A cross-sectional study was conducted between April and October 2020 in five cities, namely Yunfu, Jiangmen, Yingde, Rongcheng, and Puning, located in Guangdong Province, China. In order to prevent HIV/STD among key populations, the local Centre for Disease Control and Prevention has established outreach service teams in each city for many years. These outreach teams have long-standing relations with local FSW and can provide comprehensive health services. These teams consisted of at least one public health worker and one medical staff member who possessed extensive experience in HIV/STD intervention and outreach services. Their work encompassed various activities, including sexual health education, condom distribution, HIV/STD testing and counseling, as well as facilitating the linkage to care for FSW.

### Study participants

All participants were recruited through outreach services. To be eligible for participation, individuals had to meet the following inclusion criteria: (1) cisgender woman; (2) be 18 years of age or older; (3) have engaged in sex in exchange for goods or money at least once in the past year; (4) have the willingness to be tested for HIV/STD; (5) express willingness to participate in the survey by providing written informed consent.

### Data collection

Data collection for this study utilized paper questionnaires, which were developed through discussions with the study group, international HIV/STD experts, and local outreach service workers. To ensure the validity of the survey, a pilot test was conducted with ten FSW to assess the questionnaire items and the capacity of the local outreach team. The pilot data were not included in the final analysis.

Prior to the survey, the local outreach team conducted a mapping exercise to identify the locations of sex work venues in each site, taking into account the geographical area and types of venues. The recruitment of FSW was conducted through venue-based convenience sampling. We categorized the venues into middle-level and low-level based on the clientele’ socioeconomic status. Middle-level venues included bath centers, nightclubs, bars, dance halls, and hotels, while low-level venues encompassed foot-bathing shops, hair salons, barber shops, roadside shops, roadside restaurants, streets, and other public outdoor areas. Face-to-face survey interviews were then conducted by local outreach workers with all eligible participants at their respective working venues. The survey took approximately ten minutes to complete. All survey data were treated as anonymous and confidential, and written consent was obtained prior to the commencement of the survey.

After completing the questionnaires, participants underwent testing for HIV, syphilis, gonorrhea, and chlamydia. Venous blood samples were collected from each participant for HIV and syphilis tests. HIV screening was conducted using an enzyme-linked immunoassay (Lizhu Biotech, Zhuhai, China), which was confirmed by the local Centers for Disease Control and Prevention. Syphilis testing involved the rapid plasma reagin test (Lizhu Biotech, Zhuhai, China), which was further confirmed by the treponema pallidum particle agglutination test (Rongsheng Biotech, Shanghai, China). Both the HIV and syphilis test kits were approved by the State Food and Drug Administration of China. Testing for HIV and syphilis was carried out at local sites. All urine samples were tested for gonorrhea and chlamydia using Nucleic Acid Amplification Tests (Roche Molecular Systems, Inc. New Jersey, USA) at the Dermatology Hospital of Southern Medical University. Post-test counseling and appropriate medical care for HIV/STD were provided in accordance with the Chinese standard STD clinical management guidelines.

### Measurements

#### Social-demographic and behavioral variables

The socio-demographic information collected in this study encompassed various variables, including age, ethnicity, marital status, length of time working in the current location, highest level of education attained, and annual income. Regarding sexual behavior, the variables of interest included the number of clients in the past week and condom usage within the past month. Consistent condom use was defined as always using condoms when engaged in sex including non-clients. Vaginal discomfort was defined as vaginal pain, abnormal vaginal discharge and vulval ulcers. Additionally, reproductive health variables focused on lifetime occurrences of unintended pregnancy, infertility, and abortion.

#### Characteristics of online commercial sex-seeking

This study aimed to gather information on online commercial sex-seeking among participants, including whether they actively sought sexual partners online through platforms such as weibo, websites, blogs, and apps, as well as the frequency of their social application use. For the purposes of this study, online commercial sex-seeking was defined as utilizing any online platforms to find sexual partners in exchange for goods or money. Furthermore, long-term engagement in online commercial sex-seeking referred to the continuous use of social apps for a duration exceeding one year. In this study, STD was defined as the diagnosis of any of the following conditions: syphilis, chlamydia, gonorrhea, and HIV infection.

### Statistical analysis

A descriptive analysis was performed to provide an overview of the sociodemographic characteristics, sexual behaviors, reproductive health, prevalence of HIV/STD, and patterns of online commercial sex-seeking use. Univariate and multivariable logistic regression analyses were conducted to examine the factors associated with online commercial sex-seeking use, with adjustments made for age, legal marital status, education, and annual income in the multivariable model. Additionally, a sub-analysis was conducted to explore the correlates of long-term online commercial sex-seeking use among participants who had previously engaged in such activities. Statistical significance was defined as a P-value of < 0.05. All data analyses were conducted using SAS 9.2 (SAS Institute Inc., Cary, NC).

## Results

### Sociodemographic characteristics

Overall, 1217 women were recruited in physical venues in this survey. Sixty-two individuals declined to participant the study. Finally, a total of 1155 female sex workers, primarily from low- and middle-level venues, participated in this survey. The participants had a mean age of 33.17 ± 9.89 years. A significant proportion of the participants were under the age of 30 (40.26%, 465/1155), married (43.64%, 504/1155), had completed junior high school (53.94%, 623/1155), reported an annual income between USD 5000 and USD 10,000 (33.42%, 386/1155), and had been working in their current location for over one year (40.87%, 472/1155) (Table 1).

**Table 1:** Demographics and sexual behaviors of FSW in Guangdong Province, China, 2020 (*N* = 1155)

Characteristics	Total *n* (%)	Online sex-seeking		*P*
		Yes *n* (%)	No *n* (%)	
**Demographics**	**1155**	**386(33.42)**	**769(66.58)**	
**Age (Year)**				< 0.001
**Mean ± SD**	33.17 **±** 9.89	34.86 **±** 10.56	32.32 **±** 9.43	
<=30	465(40.26)	130(33.68)	335(43.56)	
30–40	434(37.58)	145(37.56)	289(37.58)	
> 40	256(22.16)	111(28.76)	145(18.86)	
**Ethnicity**				0.147
Han	1059(91.69)	347(89.90)	712(92.59)	
Non-Han	96(8.31)	39(10.10)	57(7.41)	
**Marital status**				0.620
Married	504(43.64)	164(42.49)	340(44.21)	
Unmarried	651(56.36)	222(57.51)	429(55.79)	
**Highest education**				0.125
Primary school or less	250(21.65)	77(19.95)	173(22.49)	
Junior high School	623(53.94)	201(52.07)	422(54.88)	
Senior high school and above	282(24.42)	108(27.98)	174(22.63)	
**Annual income ($)**				< 0.001
< 5000	177(15.32)	39(10.10)	138(17.95)	
5000–10,000	386(33.42)	103(26.68)	283(36.80)	
10,001–15,000	345(29.87)	120(31.09)	225(29.26)	
> 15,000	247(21.39)	124(32.12)	123(15.99)	
**Workplace**				0.073
Middle tier	690(59.74)	216(55.96)	474(61.64)	
Low tier	465(40.26)	170(44.04)	295(38.36)	
**Length of time working in current location (month)**				0.221
< 6	356(30.82)	107(27.72)	249(32.38)	
6–12	327(28.31)	110(28.50)	217(28.22)	
> 12	472(40.87)	169(43.78)	303(39.40)	
**Vaginal discomfort**				0.951
No	739(63.98)	246(63.73)	493(64.11)	
Yes	416(36.02)	140(36.27)	276(35.89)	
**Received health services locally**				< 0.001
No	275(23.81)	49(12.69)	226(29.39)	
Yes	880(76.19)	337(87.31)	543(70.61)	
**Sexual Behavior**				
**Number of clients in the last week**				< 0.001
**Mean ± SD**	7.92 ± 7.23	10.06 ± 8.50	6.84 ± 6.24	
<=7	667(57.75)	161(41.71)	506(65.80)	
8–14	307(26.58)	144(37.31)	163(21.20)	
>=15	181(15.67)	81(20.98)	100(13.00)	
**Consistent condom uses in the last month**				0.970
No	315(27.27)	105(27.20)	210(27.31)	
Yes	840(72.73)	281(72.80)	559(72.69)	
**Reproductive health**				
**Ever had an unintended pregnancy due to commercial sex**				0.004
No	1024(88.66)	327(84.72)	697(90.64)	
Yes	131(11.34)	59(15.28)	72(9.36)	
**Ever have been diagnosed as infertile**				< 0.001
No	880(76.19)	317(82.12)	563(73.21)	
Yes	30(2.60)	15(3.89)	15(1.95)	
Unknown	245(21.21)	54(13.99)	191(24.84)	
**Ever abortions**				< 0.001
No	767(66.41)	223(57.77)	544(70.74)	
Yes	388(33.59)	163(42.23)	225(29.26)	
**STD testing results**				
**HIV positive**				
No	1154(99.91)	385(99.74)	769(100.00)	
Yes	1(0.09)	1(0.26)	0(0.00)	
**Syphilis positive**				0.436
No	1145(99.13)	381(98.70)	764(99.35)	
Yes	10(0.87)	5(1.30)	5(0.65)	
**Gonorrhea positive**				0.143
No	1118(96.80)	369(95.60)	749(97.40)	
Yes	37(3.20)	17(4.40)	20(2.60)	
**Chlamydia positive**				0.061
No	1007(87.19)	326(84.46)	681(88.56)	
Yes	148(12.81)	60(15.54)	88(11.44)	
**STD positive**				0.019
No	984(85.19)	315(81.61)	669(87.00)	
Yes	171(14.81)	71(18.39)	100(13.00)	
**Lifetime history of testing**				
**Ever tested for chlamydia**				0.001
No	1051(91.00)	333(86.27)	718(93.37)	
Yes	104(9.00)	53(13.73)	51(6.63)	
**Ever tested for gonorrhea**				0.001
No	1071(92.73)	344(89.12)	727(94.54)	
Yes	84(7.27)	42(10.88)	42(5.46)	

### Use of online commercial sex-seeking

Among the total of 1155 participants, 33.42% (386/1155) reported ever using the internet to seek commercial sex. A comparison between online sex-seeking users and non-online users revealed several significant differences using bivariate analyses. The online users tended to be older (*P* < 0.001), had higher annual incomes (*P* < 0.001), were more likely to access local health services (*P* < 0.001), had a higher number of clients in the past week (*P* < 0.001), had a higher proportion of unintended pregnancies (*P* = 0.004), had a higher likelihood of experiencing infertility (*P* < 0.001) and abortions (*P* < 0.001), and had a higher proportion of STD cases (*P* = 0.019) (Table 1).

Among the participants who reported ever seeking sex online, 33.94% (131/386) engaged in long-term online commercial sex-seeking. Additionally, within this group, 79.27% (306/386) had sought sexual partners online in the past week. It was found that 53.63% (207/386) of the commercial sex encounters occurred within two days of meeting the last sexual partner online. Furthermore, 61.39% (237/386) of the participants took less than 12 h from the initial interaction to the actual meeting. In terms of protective measures, 84.20% (325/386) reported using condoms with their last online sexual partner. Interestingly, 49.48% (191/386) indicated that they would discuss condom usage before the meeting. Additionally, 34.97% (135/386) of the FSW were asked about their STD status prior to meeting up (Table 2).

**Table 2:** Behaviors of FSW who ever used online to seek commercial sex in Guangdong Province, China (*N* = 386)

Variables	*n*	%
**Length of time since started to find sexual partners online**		
< 6 months	107	27.72
6 months-1 year	148	38.34
1–3 years	114	29.53
> 3 years	17	4.40
**Engaged in finding sexual partners online in the last weeks**		
No	80	20.73
Yes	306	79.27
**Number of sexual partners seeking online in the last week**		
0–3	259	67.10
4–6	99	25.65
7–10	16	4.15
> 10	12	3.11
**Length of time since met the last sexual partner online (days)**		
< 1	65	16.84
1–2	142	36.79
3–4	81	20.98
5–7	55	14.25
8–30	24	6.22
> 30	19	4.92
**The time taken from the first interaction to meeting in reality (hours)**		
< 1	49	12.69
1–12	188	48.70
13–24	100	25.91
> 24	49	12.69
**Used condoms during last sex with the last online partner**		
No	61	15.80
Yes	325	84.20
**Discussed about condom use before met**		
No	195	50.52
Yes	191	49.48
**Being asked about your STD status before meeting up**		
No	251	65.03
Yes	135	34.97

### Sexual behaviors, reproductive health and prevalence of HIV/STD

Out of the 1155 individuals, the average number of clients in the last week was 7.92 ± 7.23. The majority of participants reported consistent condom use during sexual encounters in the past month (72.73%, 840/1155). A small percentage of participants (11.34%, 131/1155) reported experiencing unintended pregnancies due to commercial sex, while 33.59% (388/1155) reported having had abortions. Only a minority of participants ever had chlamydia (9.00%,104/1155) and gonorrhea (7.27%,84/1155) testing, and (2.60%, 30/1155) had been diagnosed with infertility. The prevalence of HIV, syphilis, gonorrhea, chlamydia, and STD among FSW who practice in physical venues was 0.09% (1/1155), 0.87% (10/1155), 3.2% (37/1155), 12.81% (148/1155), and 14.81% (171/1155), respectively (Table 1).

Among those FSW who also meet clients online, the prevalence of HIV, syphilis, gonorrhea, chlamydia, and STD was 0.26% (1/386), 1.30% (5/386), 4.40% (17/386), 15.54% (60/386), and 18.39% (71/386), respectively. Each of these prevalence was higher compared to FSW who had never sought commercial sex online (Table 1).

### Factors associated with online sex-seeking usage

After adjusting for age, legal marital status, education, and annual income, the multivariable logistic analysis revealed several significant correlations with online commercial sex-seeking among FSW. FSW who were older (adjusted odds ratio (a*OR)* = 2.55, 95%CI: 1.79–3.65), had higher annual income (a*OR* = 3.92, 95%CI: 2.47–6.23), reported receiving health services locally (a*OR* = 2.57, 95%CI: 1.80–3.67), had a higher number of clients in the last week (a*OR* = 2.25, 95%CI: 1.66–3.06), had experienced unintended pregnancies due to commercial sex (a*OR* = 1.78, 95%CI: 1.21–2.62), had been diagnosed as infertile (a*OR* = 3.20, 95%CI: 1.42–7.21), had a history of abortions (a*OR* = 1.69, 95%CI: 1.29–2.20), and had a history of STD (a*OR* = 1.48, 95%CI: 1.05–2.09) were found to be positively associated with online commercial sex-seeking. Additionally, FSW who underwent chlamydia testing (a*OR* = 2.42, 95%CI: 1.58–3.70) and gonorrhea testing (a*OR* = 2.60, 95%CI: 1.63–4.15) demonstrated a positive correlation with online commercial sex-seeking (Table 3).

**Table 3:** Factors associated with online commercial sex-seeking among FSW in Guangdong Province, China, 2020 (*N* = 1155)

Characteristics	cOR(95%CI)	*P*-value	aOR(95%CI) ^#^	*P*-value
**Demographics**				
**Age (Year)**				
<=30	*Ref*	-	*Ref*	-
30–40	1.29(0.97,1.72)	0.077	1.51(1.09,2.08)	0.018*
> 40	1.97(1.43,2.72)	< 0.001*	2.55(1.79,3.65)	< 0.001*
**Marital status**				
Married	*Ref*	-	*Ref*	-
Unmarried	1.07(0.84,1.37)	0.577	1.15(0.87,1.52)	0.322
**Highest education**				
Primary school or less	*Ref*	-	*Ref*	-
Junior high school	1.07(0.78,1.47)	0.675	0.93(0.66,1.32)	0.692
Senior high school and above	1.40(0.97,2.00)	0.070	1.09(0.72,1.65)	0.690
**Annual income ($)**				
< 5000	*Ref*	-	*Ref*	-
5000–10,000	1.29(0.85,1.96)	0.239	1.25(0.81,1.91)	0.309
10,001–15,000	1.89(1.24,2.87)	0.003*	1.71(1.10,2.67)	0.018*
> 15,000	3.57(2.31,5.51)	< 0.001*	3.92(2.47,6.23)	< 0.001*
**Workplace**				
Middle tier	*Ref*	-	*Ref*	-
Low tier	1.27(0.99,1.62)	0.064	1.36(1.04,1.79)	0.093
**Ethnicity**				
Han	*Ref*	-	*Ref*	-
Non-Han	1.40(0.91,2.14)	0.119	1.12(0.72,1.75)	0.614
**Length of time working in current location (month)**				
< 6	*Ref*	-	*Ref*	-
6–12	1.18(0.86,1.63)	0.315	1.11(0.79,1.56)	0.541
> 12	1.30(0.97,1.74)	0.083	1.15(0.83,1.59)	0.405
**Vaginal discomfort**				
Yes	*Ref*	-	*Ref*	-
No	0.98(0.76,1.27)	0.900	0.83(0.63,1.08)	0.169
**Received health services locally**				
No	*Ref*	-	*Ref*	-
Yes	2.86(2.04,4.01)	< 0.001*	2.57(1.80,3.67)	< 0.001*
**Sexual Behavior**				
**Number of clients in the last week**				
<=7	*Ref*	-	*Ref*	-
8–14	2.78(2.09,3.70)	< 0.001*	2.25(1.66,3.06)	< 0.001*
>=15	2.55(1.81,3.59)	< 0.001*	2.09(1.46,3.00)	< 0.001*
**Consistent condom uses in the last month**				
No	*Ref*	-	*Ref*	-
Yes	1.01(0.76,1.32)	0.970	0.75(0.56,1.01)	0.058
**Reproductive health**				
**Ever had an unintended pregnancy due to commercial sex**				
No	*Ref*	-	*Ref*	-
Yes	1.75(1.21,2.53)	0.003*	1.78(1.21,2.62)	0.003*
**Have been diagnosed as infertile**				
No	1.99(1.43,2.78)	< 0.001*	1.57(1.10,2.23)	0.013*
Yes	3.54(1.63,7.69)	0.001*	3.20(1.42,7.21)	0.005*
Unknown	*Ref*	-	*Ref*	-
**Ever abortions**				
No	*Ref*	-	*Ref*	-
Yes	1.77(1.37,2.28)	< 0.001*	1.69(1.29,2.20)	< 0.001*
**STD testing results**				
**Syphilis positive**				
No	*Ref*	-	*Ref*	-
Yes	2.01(0.58,9.67)	0.274	2.67(0.75,9.52)	0.131
**Gonorrhea positive**				
No	*Ref*	-	*Ref*	-
Yes	1.73(0.89,3.33)	0.104	1.38(0.69,2.76)	0.361
**Chlamydia positive**				
No	*Ref*	-	*Ref*	-
Yes	1.42(1.00,2.03)	0.050	1.37(0.95,1.97)	0.096
**STD positive**				
No	*Ref*	-	*Ref*	-
Yes	1.51(1.08,2.10)	0.015*	1.48(1.05,2.09)	0.026*
**Lifetime history of testing**				
**Ever tested for chlamydia**				
No	*Ref*	-	*Ref*	-
Yes	2.24(1.49,3.36)	< 0.001*	2.42(1.58,3.70)	< 0.001*
**Ever tested for gonorrhea**				
No	*Ref*	-	*Ref*	-
Yes	2.11(1.35,3.30)	0.001*	2.60(1.63,4.15)	< 0.001*

Note: #Age, legal marital status, education and annual income were adjusted for each other, and all other variables were adjusted for age, legal marital status, education and annual income

* *P* < 0.05

### Factors associated with long-term online sex-seeking usage

After adjusting for age, legal marital status, education, and annual income, the multivariable logistic analysis revealed significant correlations with long-term online commercial sex-seeking among FSW. FSW who had a senior high school degree and above (a*OR* = 7.94, 95%CI: 3.47–18.18), had a longer working duration at their current location (a*OR* = 13.25, 95%CI: 5.89–29.81), had fewer clients in the last week (a*OR* = 0.49, 95%CI: 0.28–0.86), did not experience vaginal discomfort (a*OR* = 3.29, 95%CI: 1.89–5.74), consistently used condoms in the last month (a*OR* = 3.83, 95%CI: 2.00-7.34), and had a history of abortions (a*OR* = 1.93, 95%CI: 1.18–3.16) demonstrated positive associations with long-term online commercial sex-seeking (Table 4).

**Table 4:** Factors associated with long-term online commercial sex-seeking among FSW who ever used online to seek sex in Guangdong Province, China (*N* = 386)

Characteristics	Total *n* (%)	Short term use *n* (%)	Long-term use *n* (%)	c*OR*(95%CI)	a*OR*(95%CI) #
**Demographics**	**386**	**255(66.06)**	**131(33.94)**		
**Age (Year)**					
<=30	130(33.68)	94(36.86)	36(27.48)	*Ref*	*Ref*
30–40	145(37.56)	85(33.33)	60(45.80)	1.84(1.11,3.06) *	1.62(0.88,3.00)
> 40	111(28.76)	76(29.80)	35(26.72)	1.20(0.69,2.09)	1.63(0.83,3.19)
**Marital status**					
Married	164(42.49)	93(36.47)	71(54.20)	*Ref*	*Ref*
Unmarried	222(57.51)	162(63.53)	60(45.80)	0.49(0.32,0.74) *	0.78(0.47,1.30)
**Highest education**					
Primary school or less	77(19.95)	65(25.49)	12(9.16)	*Ref*	*Ref*
Junior high school	201(52.07)	146(57.25)	55(41.98)	2.04(1.02,4.07) *	1.73(0.82,3.64)
Senior high school and above	108(27.98)	44(17.25)	64(48.85)	7.88(3.81,16.28) *	7.94(3.47,18.18) *
**Annual income ($)**					
< 5000	39(10.10)	26(10.20)	13(9.92)	*Ref*	*Ref*
5000–10,000	103(26.68)	86(33.73)	17(12.98)	0.40(0.17,0.92) *	0.36(0.14,0.88) *
10,001–15,000	120(31.09)	57(22.35)	63(48.09)	2.21(1.04,4.71) *	1.64(0.73,3.70)
> 15,000	124(32.12)	86(33.73)	38(29.01)	0.88(0.41,1.90)	0.60(0.25,1.46)
**Workplace**					
Middle tier	216(55.96)	157(61.57)	59(45.04)	*Ref*	*Ref*
Low tier	170(44.04)	98(38.43)	72(54.96)	1.96(1.28,3.00) *	2.35(1.37,4.03) *
**Ethnicity**					
Han	347(89.90)	236(92.55)	111(84.73)	*Ref*	*Ref*
Non-Han	39(10.10)	19(7.45)	20(15.27)	2.24(1.15,4.36) *	2.11(1.01,4.42) *
**Length of time working in current location (month)**					
< 6	107(27.72)	98(38.43)	9(6.87)	*Ref*	*Ref*
6–12	110(28.50)	88(34.51)	22(16.79)	2.72(1.19,6.23) *	2.89(1.18,7.07) *
> 12	169(43.78)	69(27.06)	100(76.34)	15.78(7.47,33.36) *	13.25(5.89,29.81) *
**Vaginal discomfort**					
Yes	140(36.27)	112(43.92)	28(21.37)	*Ref*	*Ref*
No	246(63.73)	143(56.08)	103(78.63)	2.88(1.77,4.68) *	3.29(1.89,5.74) *
**Received health services locally**					
No	49(12.69)	36(14.12)	13(9.92)	*Ref*	*Ref*
Yes	337(87.31)	219(85.88)	118(90.08)	1.49(0.76,2.92)	1.01(0.46,2.20)
**Sexual Behavior**					
**Number of clients in the last week**					
<=7	161(41.71)	83(32.55)	78(59.54)	*Ref*	*Ref*
8–14	144(37.31)	106(41.57)	38(29.01)	0.38(0.24,0.62) *	0.49(0.28,0.86) *
>=15	81(20.98)	66(25.88)	15(11.45)	0.24(0.13,0.46) *	0.27(0.13,0.55) *
**Consistent condom uses in the last month**					
No	105(27.20)	90(35.29)	15(11.45)	*Ref*	*Ref*
Yes	281(72.80)	165(64.71)	116(88.55)	4.22(2.32,7.65) *	3.83(2.00,7.34) *
**Reproductive health**					
**Ever had an unintended pregnancy due to commercial sex**					
No	327(84.72)	212(83.14)	115(87.79)	*Ref*	*Ref*
Yes	59(15.28)	43(16.86)	16(12.21)	0.69(0.37,1.27)	0.86(0.43,1.69)
**Have been diagnosed as infertile**					
No	317(82.12)	193(75.69)	124(94.66)	8.03(3.18,27.06) *	8.55(3.25,29.58) *
Yes	15(3.89)	12(4.71)	3(2.29)	3.12(0.56,16.09)	3.73(0.61,21.03)
Unknown	54(13.99)	50(19.61)	4(3.05)	*Ref*	*Ref*
**Ever abortions**					
No	223(57.77)	157(61.57)	66(50.38)	*Ref*	*Ref*
Yes	163(42.23)	98(38.43)	65(49.62)	1.58(1.03,2.42) *	1.93(1.18,3.16) *
**STD testing results**					
**HIV positive**					
No	385(99.74)	254(99.61)	131(100.0)	-	-
Yes	1(0.26)	1(0.39)	0(0.0)	-	-
**Syphilis positive**					
No	381(98.70)	250(98.04)	131(100.0)	-	-
Yes	5(1.30)	5(1.96)	0(0.0)	-	-
**Gonorrhea positive**					
No	369(95.6)	242(94.90)	127(96.95)	*Ref*	*Ref*
Yes	17(4.40)	13(5.10)	4(3.05)	0.59(0.19,1.84)	0.77(0.22,2.62)
**Chlamydia positive**					
No	326(84.46)	219(85.88)	107(81.68)	*Ref*	*Ref*
Yes	60(15.54)	36(14.12)	24(18.32)	1.36(0.78,2.40)	1.67(0.87,3.20)
**STD positive**					
No	315(81.61)	209(81.96)	106(80.92)	*Ref*	*Ref*
Yes	71(18.39)	46(18.04)	25(19.08)	1.07(0.62,1.84)	1.27(0.68,2.37)
**Lifetime history of testing**					
**Ever tested for chlamydia**					
No	333(86.27)	211(82.75)	122(93.13)	*Ref*	*Ref*
Yes	53(13.73)	44(17.25)	9(6.87)	0.35(0.17,0.75) *	0.40(0.18,0.91) *
**Ever tested for gonorrhea**					
No	344(89.12)	221(86.67)	123(93.89)	*Ref*	*Ref*
Yes	42(10.88)	34(13.33)	8(6.11)	0.42(0.19,0.94) *	0.60(0.25,1.45) *

Note: #Age, legal marital status, education and annual income were adjusted for each other, and all other variables were adjusted for age, legal marital status, education and annual income

* *P* < 0.05

## Discussion

Worldwide, FSW are a group at high risk for STD exposure. [1]. Online commercial sex-seeking further contributes to the escalating STD epidemic among FSW [13]. Our study aimed to shed light on the prevalence of online commercial sex-seeking among Chinese FSW and explore associated factors, thus expanding the existing literature on this topic. The findings of our study have implications for future interventions for people who seek commercial sex partners online.

Our study revealed a high prevalence (33.4%) of online commercial sex-seeking among FSW in China, which aligns with findings from Canada [13] and Sweden [21], but exceeds the reported rates in Kosovo [14] and England [22]. This high prevalence may be attributed to the widespread popularity and discreet nature of the internet, which facilitates the ease of finding sexual partners. Despite efforts in many countries, including China, to restrict or monitor online sex markets (such as combating online sex trafficking and regulating sex advertising) [23], the impact of these measures remains limited. Notably, the internet has been widely utilized as a platform for delivering interventions aimed at promoting health-related behaviors on a global scale [24]. Therefore, it is crucial to consider internet-based health promotion and risk reduction interventions specifically tailored to FSW, such as disseminating sexual health text messages and risk assessments through social media, providing HIV/STD testing services, and establishing online platforms for FSW to consult with healthcare professionals [25].

Our findings highlight that FSW engaging in online commercial sex-seeking are at a higher risk of experiencing reproductive health issues, especially for unintended pregnancies and abortions. Abortion is legal and is an essential component of women’s reproductive health care in China. This finding that has not been consistently observed in previous studies. Several factors may contribute to this association. Firstly, online users are more likely to engage in condomless sex than FSW who had never sought commercial sex online [15–17]. Secondly, FSW often find themselves marginalized in humanitarian contexts, resulting in their reproductive health needs being overlooked [26]. Lastly, the internet provides FSW seeking commercial sex online with increased anonymity and mobility, which can hinder healthcare providers from delivering essential health and social services to this population [27]. As online commercial sex-seeking continues to gain popularity among FSW [28], healthcare practitioners should adopt harm reduction approaches and develop innovative interventions that promote the safe use of the internet to address reproductive health concerns [17]. However, it is worth noting that existing interventions among online users primarily focus on HIV/STD prevention and individual behavior change [28], with relatively less attention given to addressing reproductive health needs [1, 29]. Therefore, we recommend the provision of comprehensive sexual and reproductive health services, including the prevention of unintended pregnancies, cervical cancer screening, and safe abortion services, specifically tailored to FSW who seek commercial sex online.

We observed alarmingly low rates of chlamydia and gonorrhea testing among the general population of FSW. Although we found that online commercial sex-seeking FSW exhibited slightly higher testing rates compared to the FSW who had never sought commercial sex online, only a small fraction of online users had ever undergone chlamydia and gonorrhea testing in their lifetime. Several factors likely contribute to these low testing rates among FSW. Firstly, the perception of STD testing as well as the fear of stigma and discrimination from healthcare providers play a role in deterring FSW from seeking testing services [30]. Secondly, the inconvenience, lack of privacy, and limited accessibility of facility-based chlamydia and gonorrhea testing further contribute to the low uptake. Lastly, for online users, the secretive and mobile nature of their online commercial sexual activities may impede healthcare providers from offering STD testing services [27]. In terms of HIV testing, there is valuable experience to draw upon in order to facilitate testing. The World Health Organization recommends innovative strategies such as social network/internet-based HIV self-testing or home-based self-collection to expand HIV testing services among hidden key populations [31]. Previous studies have demonstrated the effectiveness of these strategies in significantly increasing HIV testing rates [32, 33]. Therefore, considering the characteristics of Chinese FSW, similar approaches could be adapted to improve chlamydia and gonorrhea testing among this population.

This study has a few limitations worth mentioning. Firstly, the data on online commercial sex-seeking and sexual behaviors were collected through voluntary self-reporting, which may introduce information bias. Secondly, as this was a cross-sectional study, the observed correlations between online commercial sex-seeking and STD or reproductive health outcomes should be interpreted as associations rather than establishing causation. Thirdly, the recruitment of FSW in this study was not randomized and was limited to cities with extensive experience in STD prevention, which may restrict the generalizability of the findings to cities with limited experience. Fourthly, although the study is focused on FSW who advertise and solicit online, all the participants were drawn from physical venues, the FSW who only solicit online were not included in the study. Lastly, since all data were collected through anonymous paper questionnaires, it could be possible to accidentally have the same people participate multiple times.

## Conclusions

In our study, we observed a high prevalence of online commercial sex-seeking among FSW in China. This behavior was found to be positively associated with risks related to STD and reproductive health. Considering the widespread prevalence and significant implications of online commercial sex-seeking, it is crucial to develop targeted interventions that address healthcare needs and reproductive health services specifically for FSW.

## Data Availability

The dataset used in the study are available from the corresponding author on reasonable request.

## List of abbreviations

FSW: Female sex workers
STD: Sexually transmitted diseases
HIV: Human immunodeficiency virus
aOR: Adjusted odds ratio
